# Diagnosis of Ocular Loiasis in a Patient from a *Dirofilaria*-Endemic Area

**DOI:** 10.1590/0037-8682-0343-2021

**Published:** 2021-08-20

**Authors:** Anil Kumar, Nandita Shashindran

**Affiliations:** 1 Amrita Institute of Medical Sciences, Amrita Vishwa Vidyapeetham, Ponekara, Kochi, Kerala India.

A 49-year-old man visited our hospital to confirm filarial infection. He had a history of subconjunctival worm extraction in Dubai, where the peripheral blood smear was positive for microfilaria. He was then referred for treatment in his hometown (Kerala, India). He had no systemic or visual symptoms. He had worked in Nigeria for three years before moving to Dubai. Histopathological sections and peripheral blood smears were analyzed. Longitudinal sections of the extracted adult worm showed a rounded anterior end with no lips (Figure 1A) and the uterus filled with embryonic microfilariae (Figure 1B). Blood smears revealed sheathed microfilariae, with nuclei extending to the pointed tail tip (Figure 1C, 1D). The adult worm was identified as a female *Loa loa* worm. He received systemic therapy with corticosteroids and diethylcarbamazine. 

**FIGURE 1:**
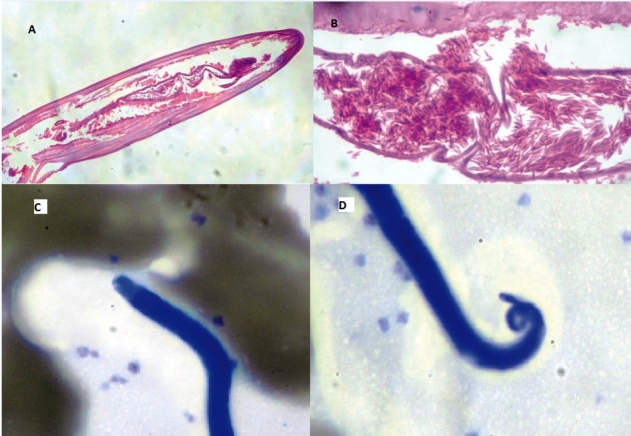
Longitudinal sections of the adult worm showing a rounded anterior end with no lips **(A)** and the uterus filled with embryonic microfilariae **(B)**. The exterior region of the cuticle lacks ridging as seen in*Dirofilaria* *repens*. Giemsa-stained microfilaria of*Loa loa*in a thin blood smear showing a cephalic space **(C)** and nuclei extending to the pointed tip of the tail **(D).**

*Dirofilaria repens* and *Brugia malayi* infections are endemic to Kerala^1^. Subconjunctival dirofilariasis is very common in Kerala and needs differentiation from loiasis, which has a similar presentation^1^ ^,^ ^2^. Our patient most likely acquired the infection in Nigeria, where *Loa loa* is endemic^3^. The adult worm of *D. repens* can be differentiated from *Loa loa* based on longitudinal ridges on the cuticle and that it is rarely detected in the gravid state^2^. Except for a few cases, microfilaremia has not been reported in human dirofilariasis^1^. The sheathed microfilaria of *B. malayi* has a hot-pink sheath (Giemsa stain) and two terminal nuclei in the blunt tail tip, whereas *Loa loa* has a colorless sheath (Giemsa stain), dense nuclear column, and nuclei extending to the pointed tip^2^. However, the microfilaria of *Dirofilaria* spp. and *Loa loa* is quite similar^2^. Correct identification can help in deciding the appropriate treatment modality. For human dirofilariasis, treatment by surgical excision of the worm without systemic antiparasitic drugs is sufficient, whereas for *B. malayi* infections, systemic antiparasitic drugs are curative. However, loiasis treatment requires both surgical excision and systemic antiparasitic drugs^3^. 

## References

[1] Sabu L, Devada K, Subramanian H (2005). Dirofilariosis in dogs and humans in Kerala. Indian J Med Res.

[2] Mathison BA, Couturier MR, Pritt BS (2019). Diagnostic Identification and Differentiation of Microfilariae. J Clin Microbiol.

[3] Emukah E, Rakers LJ, Kahansim B, Miri ES, Nwoke BEB, Griswold E (2018). In Southern Nigeria Loa loa Blood Microfilaria Density is Very Low Even in Areas with High Prevalence of Loiasis: Results of a Survey Using the New LoaScope Technology. Am J Trop Med Hyg.

